# One hundred years of BCG: the journey of tuberculosis vaccination in Brazil

**DOI:** 10.3389/fimmu.2025.1655969

**Published:** 2025-09-30

**Authors:** Eloise Trostdorf Monteiro Filardi, Roberto Carlos Cruz Carbonell, Fernando Rogério Pavan, Felipe Augusto Cerni, Manuela Berto Pucca

**Affiliations:** 1 Graduate Program in Bioscience and Biotechonology Applied to Pharmacy, School of Pharmaceutical Sciences, São Paulo State University (UNESP), Araraquara, São Paulo, Brazil; 2 Medical School, Federal University of Roraima, Boa Vista, Brazil

**Keywords:** BCG, bacillus Calmette-Guérin, vaccine, *Mycobacterium tuberculosis*, immunization

Vaccines represent one of the most significant achievements in medical science, constituting safe preparations or substances developed with the primary purpose of stimulating the immune system (1, 2). Their central function is to protect the human body against a myriad of infectious diseases that can be prevented through immunization. From an educational perspective, vaccines can be described as biological preparations that “train” the immune system to recognize and mount an effective defense against specific pathogens (e.g., viruses or bacteria) prior to natural exposure, thereby preventing the onset of disease (3).

The relevance of vaccines transcends individual protection, extending to a considerable socioeconomic impact. When employed as a public health strategy, vaccines are widely considered one of the best investments available, primarily due to their exceptional cost-effectiveness (4). The benefits of vaccination extend far beyond the immediate prevention of disease in individuals. By enabling large-scale disease control, vaccines significantly reduce the burden on healthcare systems, leading to fewer hospital admissions, diminished reliance on high-cost medical interventions, and reduced demand for long-term care services. Moreover, individuals protected through immunization are generally healthier and more capable of contributing to the workforce, thereby enhancing economic productivity at both community and national levels (5, 6).

The history of vaccination is a narrative of scientific triumphs and transformative impact on global health. Over the past five decades, global immunization efforts have resulted in an extraordinary achievement. It is estimated that at least 154 million lives have been saved, the vast majority of them (approximately 101 million) being children (7). A particularly eloquent testament to this success is the impact of measles vaccination, which alone accounted for about 60% of those lives saved, equivalent to approximately 94 million children protected since 1974 (8, 9).

This year marks a significant milestone, a century of the Bacillus Calmette–Guérin (BCG) vaccination in Brazil, a pivotal tool in the fight against tuberculosis (TB), a disease responsible for thousands of deaths worldwide. Officially introduced in Brazil in 1925, the BCG vaccine became a symbol of prevention and public health advancement against one of the most lethal infectious diseases globally (10, 11). However, the vaccine’s origin dates to 1921 (ie., four years before), when French scientists Albert Calmette and Camille Guérin, working at the Pasteur Institute in Paris, developed the immunizing agent after years of dedicated research. The scientists attenuated a strain of *Mycobacterium bovis*, a close relative of *M. tuberculosis*, the pathogen responsible for human tuberculosis. Over the course of 13 years, they subjected the bacterium to 231 serial passages in subcultures containing bile, gradually reducing its virulence while retaining its immunogenic capacity. The outcome was a live attenuated vaccine capable of inducing protective immunity without causing disease, first administered to humans in 1921 and subsequently adopted worldwide (12).

In Brazil, before the introduction of BCG vaccine (1925) and, later, the development of effective therapeutics from the mid-20th century onwards, the tuberculosis scenario in Brazil was critical. The disease, often called the “white plague,” was one of the leading causes of death, surrounded by fear and social stigma (13). In the absence of specific treatments, therapeutic approaches were largely palliative and of limited efficacy. In that time, the main strategy for managing patients consisted of isolation in sanatoria, institutions typically built in locations considered healthy climates (mountains or coast), where prolonged rest, enhanced nutrition, and exposure to fresh air, known as “climatotherapy”, were prescribed (14). Although these represented an effort to care for patients and attempt to contain disease spread, sanatoria were insufficient to meet demand and inaccessible to most of the affected population, especially the poor. The effectiveness of these measures in curing the disease was questionable, serving more as palliation and a form of segregation (15).

**Figure 1:**
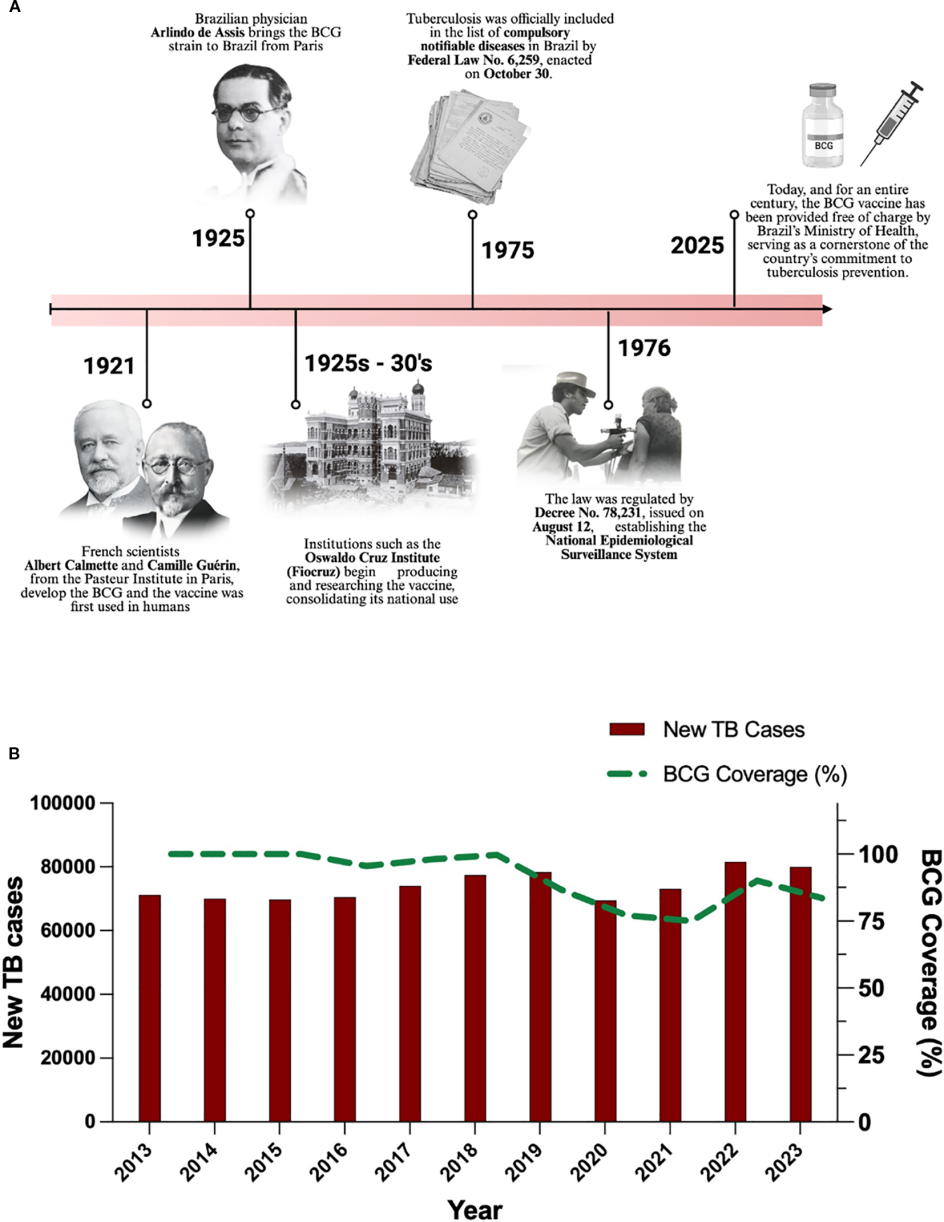
Historical and epidemiological context of BCG vaccination and tuberculosis in Brazil. **(A)** Timeline illustrating key milestones in BCG implementation in Brazil. **(B)** Temporal trends in new reported tuberculosis (TB) cases and national BCG vaccination coverage (2013–2023).

The introduction of the BCG vaccine in Brazil occurred relatively soon after its development in France, demonstrating a proactive response from the Brazilian scientific and medical community to the serious tuberculosis problem. The protagonist of this introduction was the physician and researcher Arlindo de Assis, who brought the BCG strain from Paris to Brazil in 1925 (**Figure 1A**). Linked to the Brazilian League against Tuberculosis, Assis initiated the production and application of the vaccine in the country, initially via the oral route, a method he advocated for many years (16). Subsequent production and research efforts involved renowned institutions such as the Oswaldo Cruz Institute (Fiocruz) (17), consolidating the vaccine’s presence in the national scenario from the late 1920s and early 1930s. Brazil’s early engagement with BCG technology, facilitated by figures like Arlindo de Assis and institutions like Fiocruz, signaled recognition of the magnitude of the TB problem and an openness to incorporating new scientific interventions, laying the foundation for the future integration of BCG into national immunization programs. Despite significant progress in the fight against TB, the disease remains a major global health challenge. The variable efficacy of BCG vaccine, particularly in adults, and the increasing prevalence of *M. tuberculosis* strains resistant to first-line drugs are critical concerns (18). These biomedical challenges are further compounded by persistent social determinants of health, including overcrowded living conditions, malnutrition, and limited access to timely diagnosis and effective treatment in underserved populations (19, 20).

Another important historical milestone in Brazil occurred in 1975, when tuberculosis was officially included among the diseases subject to compulsory notification. This was established by Federal Law No. 6,259, enacted on October 30, 1975, which organized the national system of epidemiological surveillance. The law was later regulated by Decree No. 78,231 of August 12, 1976 (21, 22). From that point on, all diagnosed cases of tuberculosis had to be reported to health authorities. This measure played a crucial role in strengthening public health efforts, allowing for more accurate monitoring of the disease, improved resource allocation, and the implementation of more effective tuberculosis control strategies nationwide (23–25).

Although TB is a preventable and curable disease, it remains one of the leading causes of death from infectious diseases worldwide, second only to COVID-19 during certain periods. In 2023, it is estimated that approximately 1.25 million people died from TB globally (26). In Brazil, 5,845 TB-related deaths were reported in 2022, corresponding to a mortality rate of 2.72 per 100,000 inhabitants, the highest recorded in over two decades of surveillance (27, 28). The high lethality of TB is often associated with factors such as delayed diagnosis, treatment abandonment, social vulnerability, and coinfections, particularly TB-HIV, which significantly worsens patient prognosis (29). These indicators highlight the urgent need for integrated strategies focused on surveillance, early diagnosis, treatment adherence, and public policies aimed at reducing social inequalities.

A temporal analysis of new reported tuberculosis (TB) cases in Brazil from 2013 to 2023, together with national BCG vaccination coverage, is shown in **Figure 1B**. The data demonstrate that, despite a decline in vaccination coverage during the COVID-19 pandemic, there was no immediate proportional increase in TB notifications. However, a marked rise in reported cases is observed in 2022, likely related to the resumption of surveillance activities and improved case detection.

BCG vaccination coverage in Brazil remained consistently high until 2018, with rates above 95% (**Figure 1**). From 2019 onward, a gradual decline was observed, becoming more pronounced between 2020 and 2021, when coverage dropped below 80%, despite its mandatory status under Federal Law. This decrease is likely associated with disruptions in health services, logistical challenges, and vaccine hesitancy exacerbated by the COVID-19 pandemic, as previously discussed by Filardi et al. (37). Importantly, despite the drop in coverage, no immediate proportional increase in TB cases was evident during this period. This highlights the critical need to restore and maintain high BCG vaccination coverage and to strengthen epidemiological surveillance systems to ensure timely case detection and control. Encouragingly, coverage rose to 83.5% in 2023, and preliminary data for 2024 indicate a further increase to 92.8%, suggesting a recovery toward pre-pandemic vaccination levels (30).

Vaccination remains a crucial tool in the global fight against TB, particularly given the disease’s persistent lethality and impact on vulnerable populations. The BCG vaccine, although not fully effective in preventing pulmonary TB in adults, plays a fundamental role in protecting children against the most severe and life-threatening forms of the disease, such as tuberculous meningitis and miliary TB. By reducing the incidence of these severe manifestations, BCG contributes directly to lowering TB-associated mortality and alleviating the overall burden on public health systems. Mass immunization programs, therefore, are essential to prevent avoidable deaths and to control the progression of TB, especially in high-risk communities (31, 32).

Although BCG vaccination coverage is not universal and has shown a concerning decline in several regions over recent years, it remains the most effective public health intervention for preventing severe forms of TB in children, particularly extrapulmonary manifestations. Robust evidence from observational studies and meta-analyses supports the vaccine’s efficacy, with protection ranging from 70% to 80% against these critical forms. A systematic review by the Cochrane Collaboration (2014) demonstrated a 73% reduction in the risk of tuberculous meningitis and a 77% reduction in the incidence of miliary TB among vaccinated individuals. These findings reinforce the importance of maintaining high BCG vaccination coverage as a cornerstone of global TB control strategies, particularly in endemic regions. As illustrated in the graphs for Brazil, while BCG coverage remained above 95% until 2015, a subsequent decline may jeopardize these public health gains and is potentially associated with the observed fluctuations in TB mortality. Together, these data emphasize the urgent need to strengthen immunization efforts to ensure sustained protection against severe TB and prevent a resurgence in disease-related mortality (33).

Looking ahead, scientific innovation stands as a crucial driver in the global effort to eliminate TB. Promising candidates for next-generation vaccines are in advanced stages of development, aiming to provide broader and more durable protection across age groups and geographic settings (34). In parallel, advances in molecular diagnostics and digital health technologies are enhancing early detection, while targeted public health campaigns are raising awareness and promoting adherence to treatment regimens. Together, these integrated strategies are essential for accelerating progress toward TB control and, ultimately, eradication (35, 36).

As Brazil marks a century of tuberculosis vaccination (1925–2025), this milestone serves not only as a testament to the progress achieved but also as a call to address the persistent challenges that remain. Sustained investment in public health infrastructure, research, and equitable access to healthcare, combined with ongoing scientific innovation, will be essential to ensuring that, in the coming century, tuberculosis is no longer a threat to public health in Brazil or across the globe.
