# Correction: One hundred years of BCG: the journey of tuberculosis vaccination in Brazil

**DOI:** 10.3389/fimmu.2026.1858910

**Published:** 2026-05-06

**Authors:** Eloise Trostdorf Monteiro Filardi, Roberto Carlos Cruz Carbonell, Fernando Rogério Pavan, Felipe Augusto Cerni, Manuela Berto Pucca

**Affiliations:** 1 Graduate Program in Bioscience and Biotechonology Applied to Pharmacy, School of Pharmaceutical Sciences, São Paulo State University (UNESP), Araraquara, São Paulo, Brazil; 2 Medical School, Federal University of Roraima, Boa Vista, Brazil

**Keywords:** BCG, bacillus Calmette-Guérin, vaccine, *Mycobacterium tuberculosis*, immunization

In the published article, there was a mistake in the **Funding** statement. The funder “Coordenação de Aperfeiçoamento de Pessoal de Nível Superior - Brasil (CAPES)” was erroniously omitted. The corrrect statement is:

“The author(s) declare financial support was received for the research and/or publication of this article. This study was financed in part by the Coordenação de Aperfeiçoamento de Pessoal de Nível Superior - Brasil (CAPES) - Finance Code 001. We thank Fundação de Amparo à Pesquisa do Estado de São Paulo (FAPESP; scholarship to ETMF. No. 2024/13258–0).”

The original version of this article has been updated.

